# Revisiting Early Sport Specialization: What’s the Problem?

**DOI:** 10.1177/19417381211049773

**Published:** 2021-10-15

**Authors:** Alexandra Mosher, Kevin Till, Jessica Fraser-Thomas, Joseph Baker

**Affiliations:** † York University, Toronto, Ontario, Canada; ‡ Leeds Beckett University, Leeds, England, UK

**Keywords:** adolescents, training, athlete development, specialization

## Abstract

**Context:**

The assumed risks of early specialization in sport are well known, with several international consensus statements advising against specialization in early athlete development. However, there have been recent calls for more focused research in this area.

**Evidence Acquisition:**

Research evidence from several scientific disciplines (eg, sport psychology, sports medicine, human development) were synthesized to develop a framework for practitioners working with adolescent athletes.

**Study Design:**

Narrative review.

**Level of Evidence:**

Level 4.

**Results:**

There appear to be risks associated with a highly specialized approach to athlete training, but the mechanisms driving these effects are largely unknown. Greater attention to understanding these mechanisms would help mitigate risk and develop stronger policy for athlete development. Recommendations for program modifications are provided.

**Conclusion:**

Early specialization remains an important topic for researchers and practitioners working with youth and adolescent athletes. However, more work needs to be done to provide truly evidence-based recommendations for youth athlete training.

It appears early specialization is increasing among athletes, presumably due largely to the changing nature of youth sport participation and the professionalization of youth sport.^11,33^ Interestingly, there is no consistent definition of sport specialization. One of the earliest posited definitions described sport specialization as year-round training in a single sport at the exclusion of other sport or nonsport activities.^80^ While there is some variation, researchers have found that the average age of sports specialization for elite athletes is about 14 years^10,13,70^ and is therefore during a crucial stage in human development—early adolescence. According to the World Health Organization, adolescence occurs between 10 and 19 years of age and is the transition period from childhood to adulthood.^82^ Although the age at which this life stage occurs can vary by sex (ie, girls typically reach it earlier than boys), in sport, early adolescence is usually marked by an increase in the volume of sport (ie, training and competition), and an increased pressure to specialize to become an elite athlete.^78^

The notion that earlier specialization increases the likelihood of eventually achieving elite sport performance mainly comes from research using the “deliberate practice framework.”^22^ As the name suggests, this framework emphasizes the time spent in training and proposes a monotonic relationship between hours spent engaging in effortful, domain-specific (ie, sport-specific) “deliberate practice” and performance. Even more relevant to the concept of *early* specialization, Ericsson et al^22^ suggested (1) the sooner one began deliberate practice, the sooner one would reach a high level of performance and (2) those who started deliberate practice later would not be able to reach the same level of performance as their earlier starting peers.

Despite support for other elements of this framework (eg, the positive relationship between overall time spent in training and eventual level of attainment, and the importance of domain specificity, see Young et al,^83^ for a recent review), there is a growing body of evidence suggesting early specialization is not a prerequisite for elite level attainment in sport.^10,13,30,57^ Furthermore, early specialization among youth athletes is linked to negative consequences.^31,37,43^ One of the main concerns of early specialization relates to injuries. Athletes who are highly specialized are at greater risk of serious overuse injuries^31^ and are more likely to report a previous overuse injury.^6^ In addition to these types of negative physical outcomes, there is also concern about negative effects on psychological outcomes. For example, early specialization is associated with psychological needs dissatisfaction^44^ and emotional exhaustion.^68^ Although there have been a number of consensus statements and recommendations about the dangers of early specialization,^11,18,37^ the relationship between early specialization as a behavior and these negative consequences is poorly understood.^4^

There are likely several reasons for this disconnect in understanding. First, there is a surprising lack of research on this topic of early specialization, given its prominence in discussions of youth sport and athlete development. A recent systematic review,^45^ including both empirical and nonempirical peer-reviewed papers, found that much of the literature was recirculated information in the form of commentaries and editorials. While there is value in expert opinion and summaries of previous literature, in order for the field to advance, there is a clear need for more criticality and data driven research. Of the data-driven articles, only 48 were aimed at advancing understanding of specialization in sport, and of those, only 25 examined “early” specialization.

One of the main concerns of early specialization is the outcome of overuse injuries, yet 2 separate systematic reviews and meta-analyses^5,14^ specifically evaluating specialization and overuse injury included only 5 and 6 studies, respectively. In a broader review of several aspects of specialization (eg, number of sports, months per year and hours per week of involvement, multiple team participation) and injury, only 12 studies were included. In addition to the lack of research related to specialization and injury, some have suggested that there are “substantial gaps in the scientific literature regarding the effect of specialization on motor control development, sport performance, musculoskeletal injury risk, psychosocial outcomes, burnout, attrition, and optimal strategies for youth athletes’ training and development in specific sports.”^33^ This lack of research across the field leads to a lack of understanding of specialization as a whole.

A second factor contributing to poor understanding of early specialization and potential negative consequences is the lack of a clear and consistent definition of specialization. A systematic review by Mosher et al^45^ reported a range of inconsistencies in the definitions and components used for specialization. While time spent in deliberate practice has often been suggested to be the underpinning rationale for specialization, Mosher et al^45^ found that only 9% of studies included elements of practice in their definition of specialization. Additionally, 17% of studies failed to define specialization altogether.^45^ This corroborates a 2019 review that found only 32.5% of studies operationally defined specialization.^19^ *Early* sport specialization becomes even more difficult to define as the parameters for “early” are arbitrary and change depending on both the sport and researcher. In previous work, some of these parameters have included (1) 12 years of age or earlier,^15,70^ (2) before 15 years of age,^60^ (3) before high school,^81^ and (4) as old as 23 years of age (in a sample of marathon runners.^49^ Recently, a group of researchers formed a Delphi panel and came to a consensus definition of specialization as intentional and focused participation in a single sport for the majority of the year that restricts opportunities for engagement in other sports and activities.^8^ While this is a more encompassing definition, whether it is accepted and widely used in the field remains to be seen. Until there is a concrete definition of the concept of specialization, researchers will continue to struggle to fully understand these relationships.^71^

Collectively, these first 2 factors lead to the third and arguably most substantial limitation to our understanding of the relationships between specialization and negative consequences, a lack of knowledge regarding the mechanisms underpinning these relationships. In a 2009 review of the literature, Baker et al^3^ attempted to explain the mechanisms behind specialization by suggesting a range of potential factors. Unfortunately, despite the authors’ recommendations for future research that would better explain this connection, current research has taken to using a blanket construct of “specialization” that is both inconsistently defined and unreliably measured. This has led to (1) an inability to draw cause-and-effect relationships between specialization and negative consequences and (2) an inability to design optimal training and development environments.

While these issues have clear implications for researchers, their relevance for practitioners is even more important. Practitioners are warned to advise parents and athletes against the practice of specialization without understanding *why* or *how* it should be avoided. In a multidisciplinary review that provided a broad picture of the empirical research performed on the topic of specialization, DiSanti et al^19^ summarized the work and conclusions in this area but did not provide possible explanations for these associations. As highlighted in a recent editorial by Baker et al,^4^ “We need greater attention to the mechanisms driving any negative effects. What is it about specialization that leads to negative outcomes?” Commentaries, editorials, and reviews are regularly added to the literature on specialization, but few extend our understanding of the mechanisms underpinning these negative specialization effects. Without this understanding of the processes by which these negative events occur, practitioners, parents, and other stakeholders cannot design healthy training environments to buffer against the mechanisms.

## A Framework for Exploring Early Specialization in Sport

**Figure 1.**
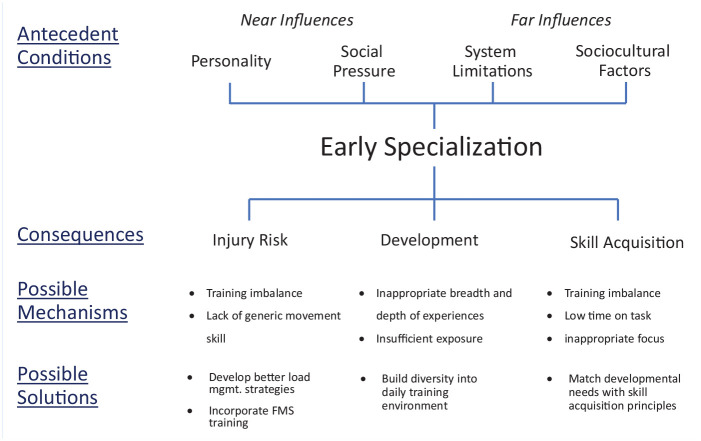
A framework for exploring early specialization in sport. Fundamental movement skills (FMS) are the basic building blocks of advanced movement that occurs later in athlete development.

In an effort to move the discussion forward, in this section, we use the existing literature on specialization to provide a framework for future work exploring these mechanisms (Figure 1).

### Antecedent Conditions

The model begins with establishing that early specialization does not happen in a vacuum. There are several antecedent conditions that promote its occurrence, which we have divided into those that have a close relationship with the athlete (*near influences*) and those occurring more distally (*far influences*). First, athlete-specific characteristics such as *personality* or motivation are near influences that may promote a more specialized focus during adolescence. For instance, athletes with high levels of passion^75^ or commitment^63^ may have a more focused engagement profile than those with lower scores on these measures. Moreover, *social pressure* from significant others such as peers, parents, or coaches could exert powerful influences on the decision to specialize in a single sport (eg, to be with key peers, to please valued coaches or parents).

In addition to these proximal variables, there are a number of more distal social and sport-related factors that can influence the likelihood of specialization. For example, one *system limitation* might be when sport funding comes from the number of enrollments in a program, resulting in programs being cautious of athletes participating in other sports. In many national sporting systems, sports are largely “siloed” and losing athletes (ie, to another sport) has significant repercussions for the short- and long-term success of the program. As a result, these sports may create additional training programs to complete in the off season to maintain athletes’ engagement within this one sport. Finally, the *sociocultural factors* associated with specific sports can promote more specialized engagement as the norm, seen most obviously in sports commonly referred to as “early specialization sports” such as gymnastics, figure skating, and diving. Surprisingly, there has been relatively little exploration of how these antecedents (and others) promote early specialization. As we note later in the paper, understanding the conditions from which a specialized athlete emerges could be valuable for understanding the most appropriate response. It is to this understanding of mechanisms and responses that we turn next.

### Consequences and Mechanisms

Based on prior work, we have noted 3 main categories of consequences associated with early sport specialization, although it is possible others will emerge after more research attention to the mechanisms driving these effects. The first category deals with the increased *injury risk* that is regularly noted as a negative outcome of specialization.^7,31^ Presumably, these increased risks are associated with inappropriate training loads leading to overuse injuries.^21^ Some have also noted that increased injury risk could come from the lack of foundational, or so-called “fundamental” movement skills. The implication here is that specialized involvement does not provide athletes with the same broad exposure to movement opportunities, which ultimately limits their experiential foundation and increases injury risk.

In addition to the obvious ill effects of chronic injuries on athletes’ physical development, other areas of *development* have been negatively associated with early specialization. For instance, early studies have suggested participation in intensive training with limited engagement with peers during early development can limit the acquisition of social skills (see Baker et al^3^ for a review). Importantly, much of this work needs to be replicated in contemporary samples. There are also links between negative psychological indicators such as eating disorders and early specialization sports,^35,69^ although this relationship may reflect elements related to the aesthetic component of these sports, rather than being a direct consequence of specialization per se. Additionally, it has been suggested that patterns of specialization are associated with burnout and/or dropout from one’s primary sport.^25,68^ However, more recent research has refuted this claim, finding no direct link between indicators of early specialization and burnout or dropout, suggesting instead meditating effects of enjoyment, competency, and autonomy.^38^ The hypothesized mechanisms driving these negative developmental effects seem to be related to the lack of opportunities to develop “normal” skills for social, emotional, and psychological coping.

The final category of consequences, and the one that has had the greatest degree of discourse, relates to *skill acquisition*. On one hand, in the past, some researchers^16^ have argued that specialized forms of engagement compromise long-term skill acquisition by undermining intrinsic forms of motivation and enjoyment. On the other hand, others (eg, Ericsson et al^22^) have noted the specificity of training-related adaptations and the relationship between deliberate practice and attainment, which seemingly justifies the need for starting focused, specific training, as early as possible. The relationship between time spent in practice and improvement/attainment is well established in other domains (eg, chess; see Ericsson et al^22^ and Newell and Rosenbloom^48^), although the requirement of an early start age in sport seems questionable.^12,42^ From a skill acquisition perspective, the impacts from early specialization have the potential to be both positive (more time on task, promoting specific performance-related adaptations) and negative (imbalance between the developmental needs of the athlete leading to injury). Much of the debate about the value of early specialization as it relates to skill acquisition comes from the inability to reconcile these potentialities.

In the next section, we propose a range of solutions for practitioners working with young athletes to try to accommodate the risks and mechanisms that may be related to negative effects from early specialization. However, it is important to emphasize some of the limitations of this evidence base as a way of stimulating further work. For instance, most of these studies are done with small samples, some quite dated, that have never been replicated or extended beyond the original study design. This is a significant issue in sport—particularly elite sport, where the developmental context is critical. Discourse in this area seems to have accepted these study findings at face value, without the normal pushing and prodding that the scientific method uses to both stabilize robust findings and eliminate elements that do not stand up to scrutiny. In the framework we have presented, there is a need for considerable additional work in all elements of the model.

## Programs to Manage the Risks

As we suggest above, the negative consequences may not lie with early sport specialization alone but rather the design, implementation, and management of an early specialization program, similar to what has been proposed within talent identification and development systems.^55^ From this perspective, managing and minimizing the negative consequences associated with early specialization involves developing practices to avoid triggering potential driving mechanisms. We propose 5 strategies for practitioners to consider to manage risks.

### Establish an “Appropriate” Environment

Practitioners need to understand the potential risks and negative consequences associated with specialization during early adolescence. Alongside, understanding the risks and negative consequences, taking responsibility for the design and implementation of their program and establishing an appropriate environment focused on promoting positive and reducing negative health consequences is key.^71^ To establish this, it is recommended the environment has clear values, expectations and day-to-day routines within the organization, which is the responsibility of all staff working with early specialization athletes.

To prevent negative *physical* consequences, practitioners must understand, place importance on and communicate the risks of early specialization to align day-to-day practices with minimizing such risks. For example, while athletes may specialize in sports, the environment can still support the development of a broad range of skills and experiences delivered in-house such as an integrative neuromuscular program (see below) and implementation of other activities (eg, within warm-ups). This can provide opportunities for the growth of fundamental movement skills while preventing overuse injuries.^47^ To prevent negative *psychosocial* consequences, implementing an integrated approach focused on the personal, social, and physical youth development rather than sporting success alone would be beneficial.^26^ Practitioners can do this by demonstrating a safe and caring environment where the person is the focus^17,74^ rather than their athletic achievements. As mentioned previously, the process of *skill acquisition* is significantly affected by a loss of motivation and enjoyment. To prevent these negative consequences, creating autonomy-supportive, mastery-oriented, and positive climates can result in less stress, greater enjoyment and more intrinsic motivation.^77,79^

Perhaps most important, avoiding the negative consequences associated with early specialization can be done by practitioners creating an environment that values the holistic development of their athletes (ie, technical, tactical, physical, psychological, social health, and performance).^73^ This places greater importance on understanding developmental principles as they relate to children and young people, the influence of growth and maturity as well as the processes of emotional and social development. The key factor here is that development is individual focused. It is the practitioner’s responsibility to establish positive training and competitive environments, and to create relationships that focus on individual athletes’ needs in addition to the long-term objectives of performance, participation, and personal development.^76^ In early adolescence especially, coaches should strive to create a challenging and enjoyable climate that focuses on development over competition and results. Moreover, other stakeholders are also important. The inclusion of parents, guardians, and supportive others can be a further strategy, acknowledging that implementation can be a challenge.^34^ Sport organizations can develop interdisciplinary support teams with specific expertise across the sport sciences, including athletic development, injury, medical, psychological, and lifestyle factors (eg, nutrition). Finally, education of all stakeholders and athletes is vital for preventing negative consequence associated with early specialization, as well as other potential negative elements associated with youth sport participation.

### Monitor and Evaluate Athletes

Having a clear approach to monitoring and evaluation can serve several purposes with adolescent athletes, including informing needs analysis and talent identification as well as evaluating the effectiveness of training programs. Furthermore, monitoring and evaluation tools could have additional value for managing athlete health during early adolescence, thereby minimizing risks of early specialization and/or the mechanisms associated with these effects over the short and longer term.^71^ Several areas have been highlighted for establishing a monitoring and evaluation tool in adolescent athletes, including athlete wellness,^61,62^ growth and maturation for measuring when the relative risk of injury may be increased or performance may be decreased,^32,41^ training load and practices, including training diaries, to establish what the athlete is doing,^51,64^ physical development,^46^ recovery,^27^ injury prevalence and mechanisms,^53^ psychosocial factors, including burnout,^52^ perfectionistic tendencies,^2^ athletic identity,^1^ and educational attainment.^54,56^ The aforementioned list alone provides a large number of areas to monitor effectively, making this a challenge for all stakeholders. Therefore, an area for future work is the establishment of valid, reliable, and practically applicable tools that can be applied in such settings without becoming additional burdens.

### Implement Integrative Neuromuscular Programs

Participation in organized sport alone does not ensure appropriate development of strength and other biomotor abilities. Therefore, the implementation of integrative neuromuscular training (see Fort-Vanmeerhaeghe et al^24^ and Lloyd et al^39^) is a strategy to manage the *physical* risks associated with early specialization. Integrative neuromuscular training is supplemental training incorporating general (eg, fundamental movements) and specific (eg, exercises targeted to motor control deficits) strength and conditioning activities (eg, resistance, dynamic stability, core focused strength, plyometric, and agility) that are designed to enhance health and skill-related components of physical fitness.^23,47^ Integrative neuromuscular training programs allow the development of concepts of athleticism (ie, “the ability to repeatedly perform a range of movements with precision and confidence in a variety of environments, which require competent levels of motor skills, strength, power, speed, agility, balance, coordination, and endurance”^40^ and are associated with enhanced athletic qualities and reductions in negative consequences, especially injury. Standardized integrative neuromuscular programs have been designed and implemented through specific warm-up protocols within some sports (eg, soccer, FIFA 11+,^58,59^ rugby union, Activate program^28,29^), which have seen injury reductions of up to 80%. However, successful implementation and compliance toward such programs requires coach, athlete and parent education and behavior to be successful, which can be difficult because of the rigidity and repetitive nature of these programs.^20,50^ Instead, other coach education and practice frameworks have been presented (eg, RAMPAGE^72^) to provide coaches with a greater degree of freedom in choosing activities within an overall framework while still emphasizing the importance of neuromuscular development. Overall, the implementation of integrative neuromuscular training is important for early specialization athletes during early adolescence to develop biomotor abilities and reduce injury risk.

### Provide Psychological Skills Training

Because of some of the suggested negative *psychological* consequences associated with early specialization, providing psychological skills training to assist adolescent athletes in acquiring psychological strategies for coping, goal setting and managing multiple demands is important.^36,66^ In alignment with other elements noted in the sections above, this recommendation positions athletes as key agents in navigating their sport experience. Providing them with coping skills, for example, may mitigate the negative effects of performance pressure, a characteristic often seen as being associated with early specialization.

Commitment to the psychological development of resilient and adaptable athletes characterized by mental capability and robustness, high self-regulation, and enduring personal excellence qualities, is critical. Furthermore, practitioners should encourage early specialization athletes to communicate honestly about how they are feeling and utilize other monitoring tools (see the Monitor and Evaluate Athletes section). Together, this information can be used to better understand the demands and stresses on athletes and potentially change training cultures in early specialization sports. Moreover, adopting this approach, where athletes feel more supported to share their feelings and concerns with coaches, parents, and peers, could be important for managing other emerging issues in high-performance sport (eg, mental health concerns^67^).

### Manage Training Practices

Programs, particularly during early adolescence, should focus on an appropriate sport-life balance.^9^ The appropriate management of training practices, including frequency, volume, and intensity of training, alongside adequate rest and recovery, could be vital for minimizing the negative consequences of early specialization. This is important not only from both a physiological and psychological perspective to balance workload and recovery to maximize training adaptations and learning but also for providing opportunities for other priorities, including social time with family and friends, academic work, and enjoying other activities. Therefore, the careful planning of training (including a balance of technical, tactical, physical, and psychological development), competition, rest, and recovery, and the promotion of other key activities of youth development (eg, social activities) is vital to maximize positive and reduce negative consequences. However, research on key stakeholders (eg, parents) has shown limited understanding of these concepts.^7^ Importantly, managing training volumes may be *easier* in early specialization athletes with fewer stakeholders (eg, coaches) than multisport athletes, a group that has been described as “organized chaos” because of the multiple stakeholders across multisports, -clubs, and -coaches.^65^

## Conclusion

In developing the above framework and recommendations, we wish to be very clear—we have many more questions than we do answers about the relationships between early specialization (and its varied definitions) and negative health and developmental outcomes. However, based on the limited existing empirical work, the various systematic and narrative reviews, as well as the editorials and position statements, we believe the framework provides a useful roadmap for future work. Furthermore, we believe the recommendations are useful guidelines given that they have general relevance for athlete development, training load management, and positive youth development generally, and happen to focus on what researchers and policy makers believe are the key factors associated with early specialization more specifically. Continued work in this area will help us refine these recommendations as causal links between behavior and effect emerge.

The consistent interest in this area provides good momentum for future work. However, we need to move beyond the simplistic correlational studies used in prior work to prospective and longitudinal designs that can track participation patterns and developmental effects in multivariate models. Moreover, having adequate comparison groups across the spectrum of participation (eg, including those with an extreme multisport participation) would extend our understanding of the optimal forms of participation for athlete skill acquisition as well as for positive and healthy development.
